# A comprehensive review on disposition kinetics and dosage of oral administration of *Andrographis paniculata*, an alternative herbal medicine, in co-treatment of coronavirus disease

**DOI:** 10.3389/fphar.2022.952660

**Published:** 2022-08-19

**Authors:** Phanit Songvut, Tawit Suriyo, Duangchit Panomvana, Nuchanart Rangkadilok, Jutamaad Satayavivad

**Affiliations:** ^1^ Laboratory of Pharmacology, Chulabhorn Research Institute, Bangkok, Thailand; ^2^ Center of Excellence on Environmental Health and Toxicology (EHT), OPS, MHESI, Bangkok, Thailand; ^3^ Translational Research Unit, Chulabhorn Research Institute, Bangkok, Thailand

**Keywords:** pharmacokinetics, *Andrographis paniculata*, andrographolide, corona virus disease (COVID-19), dose of administration

## Abstract

Coronavirus disease 2019 (COVID-19) is a present global health crisis that is driving the investigation of alternative phytomedicines for antiviral purposes. The evidence suggests that *Andrographis paniculata* crude or extract is a promising candidate for treating symptoms of severe acute respiratory syndrome coronavirus 2 (SARS-CoV-2). This review aims to consolidate the available reports on the disposition kinetics of andrographolide, a main active component of *A. paniculata*. The second objective of this review is to summarize the available reports on an appropriate oral dosage for the use of andrographolide in upper respiratory tract infections (URTIs) and other viral infectious diseases. The data were collected from the literature on absorption, distribution, biotransformation, and excretion of andrographolide, and information was also obtained from scientific databases about the use of *A. paniculata*. The finding of this review on pharmacokinetics indicates that andrographolide is slightly absorbed into the blood circulation and exhibits poor oral bioavailability, whereas its distribution process is unrestricted. In the termination phase, andrographolide preferentially undergoes biotransformation partly through phase I hydroxylation and phase II conjugation, and it is then eliminated via the renal excretion and hepatobiliary system. The key summary of the recommended dosage for andrographolide in uncomplicated URTI treatment is 30 mg/day for children and 60 mg/day for adults. The dose for adult patients with pharyngotonsillitis could be increased to 180 mg/day, but not exceed 360 mg/day. Co-treatment with *A. paniculata* in concert with the standard supportive care for influenza reduced the severity of symptoms, shortened treatment duration, and decreased the risk of developing post-influenza complications. The recommended starting dose for use in patients with mild COVID-19 is 180 mg/day of andrographolide, based on the dose used in patients experiencing a URTI with inflammation. This review is not only applicable for evaluating the appropriate doses of andrographolide for antiviral treatments but also encourages future research evaluating the effectiveness of these recommended dosages during the COVID-19 pandemic.

## Graphical Abstract

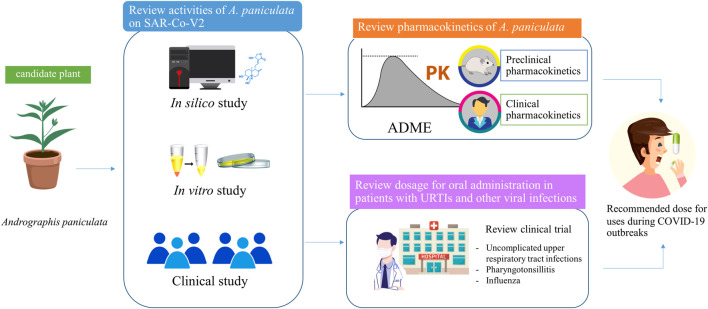

## 1 Introduction

Severe acute respiratory syndrome coronavirus 2 (SARS-CoV-2) is causing the ongoing pandemic of coronavirus disease 2019 (COVID-19). Preventing the transmission of COVID-19 is important, as new variants may continue to emerge due to the evolutionary process of coronavirus. Presently, the problem of lacking effective antiviral drugs against SARS-CoV-2 is a reason for unsatisfactory clinical outcomes in COVID-19 patients (Chan et al., 2020). Therefore, this global health crisis has driven efforts to explore alternative herbal medicines for antiviral purposes (Chaiyasit et al., 2021). The present pharmacological evidence points to *Andrographis paniculata* (Burm. f) Nees (Acanthaceae)*,* a plant that has been commonly used as traditional medicine (Jayakumar et al., 2013; Gupta et al., 2017; Sa-Ngiamsuntorn et al., 2021; Zhang H. et al., 2021), as a promising antiviral candidate for the inhibition of SARS-CoV-2 activity in Thailand. *A. paniculata* has a high safety margin for symptomatic relief of common colds and upper respiratory tract infections (URTIs) (Hancke et al., 1995; Coon and Ernst, 2004; Hu et al., 2017; Hossain et al., 2021). The World Health Organization (WHO) has summarized the applications of *A. paniculata* based on its clinical uses, which include prophylaxis and treatment of the common cold, uncomplicated sinusitis, bronchitis, and pharyngotonsillitis (World Health Organization, 2004; Hossain et al., 2021). In addition, the indications for this plant have subsequently been expanded to cover its use in patients with influenza (Chuthaputti et al., 2007).

Although the evidence supports the possible selection of *A. paniculata* as an alternative herbal medicine for SARS-CoV-2 treatment, there is a lack of comparative data on the effective doses for its application during the COVID-19 crisis. To derive the dose recommendations, present data from preclinical and clinical studies are taken into consideration. A comprehensive inquiry should include the prediction of a pharmacologically active dose and full pharmacokinetic investigations. This review, therefore, aims 1) to provide concise information regarding the disposition kinetics of a major component in *A. paniculata* extract (APE)*,* focusing on andrographolide (AP); and 2) to review the oral administration doses for its application in URTIs and other viral infectious diseases.

## 2 Literature search and article review

**FIGURE 1:**
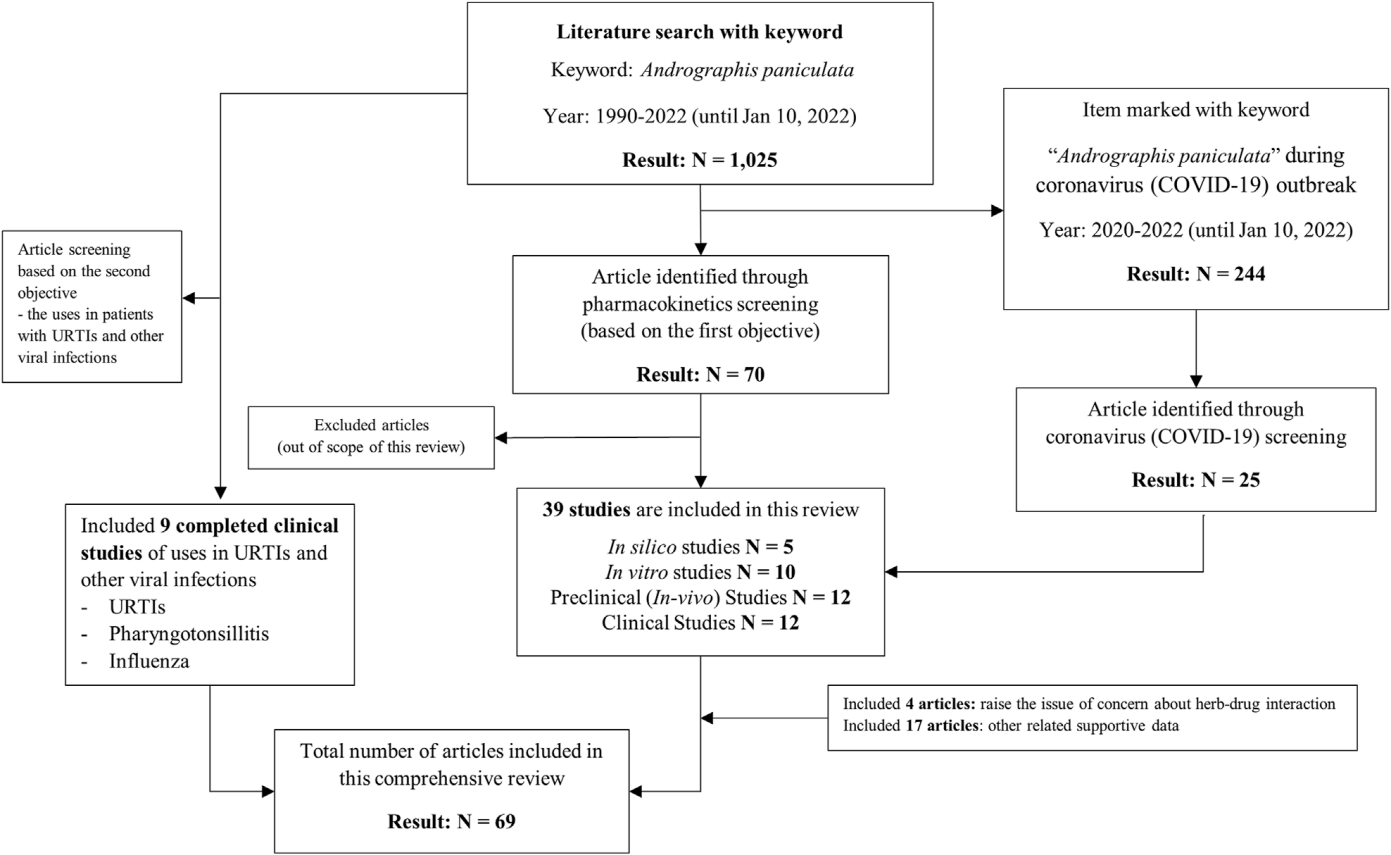
Article review of the flow chart.

A literature search was conducted in Pubmed using the keywords “*Andrographis paniculata,*” which elicited a total of 1,025 results from the years 1990–2022. These results included 244 publications during 2020–2022, after the World Health Organization (WHO) had declared the novel coronavirus (COVID-19) outbreak a pandemic (Al-Mandhari et al., 2020). A total of 25 articles searched among these 244 publications were related to the treatment of SARS-CoV-2. Through a pharmacokinetics screening, 70 articles were identified. Articles focused on the dosage of *A. paniculata* and its extract were included in the literature review. An advanced search focused on the evidence for clinical effectiveness in the relief of symptoms in patients with URTIs, pharyngotonsillitis, and influenza. A total of 69 articles related to the scope of this review were included according to the flow chart diagram shown in Figure 1.

## 3 Selection of chemical markers of *Andrographis paniculata* for herbal medicine uses

According to the literature review of the major components of *A. paniculata*, an assessment report of this plant summarized by the European Medicines Agency (Biró-Sándor, 2014) and the WHO monographs on selected medicinal plants (World Health Organization, 2004) indicated that diterpene lactones are the main constituents of *A. paniculata*. These lactones include andrographolide, deoxyandrographolide, 11,12-didehydro-14-deoxyandrographolide, neoandrographolide, andrographiside, deoxyandrographiside, and andropanoside. Different types of extracts can influence the components in herbal products. The major components would be responsible for the holistic effects of this plant, contributing to its beneficial therapeutic outcomes. To integrate the data from several studies that used different types of extracts, variations in major compounds are considered and compared in Table 1.

**TABLE 1:** Comparison of the major compounds of methanolic extract, ethanolic extract, aqueous extract, and crude powder of *A. paniculata*.

Type of extracts	Compounds	Plant material	Aims of the study	Dosing	References
Methanolic extract					
Methanolic extract	• andrographolide (AP)	Powdered dried leaves of *A. paniculata*	Absorption: plasma concentration time profiles	1 g/kg in rats (*in vivo*)	Akowuah et al. (2009)
	• 14-deoxy-11,12-didehydroandrographolide				
Ethanolic extract					
60% ethanolic water-soluble extract	andrographolide (AP)	N/A	Pharmacokinetics in rats and humans	1 mg/kg, i.v.	Panossian et al. (2000)
				20 mg/kg, p.o.	
				200 mg/kg, p.o.	
Ethanolic extract	N/A	Powdered plant material	Effect of *A. paniculata* on proinflammatory chemokine RANTES secretion in human bronchial epithelial cells infected with influenza A virus H1N1	N/A	Ko et al. (2006)
Ethanolic extract	22 tentative compounds in the extract	N/A	Metabolism of *A.paniculata* in rats	80 g/kg, p.o. in rats	Li et al. (2015)
	Skullcapflavone I; 3α,15,19-Trihydroxy-8 (17),13-ent-labdadien-16-oic acid; Andrographidine B; 3,18,19-Trihydroxy-ent-labda-8 (17),13-dien-16,15-olide; 12-Hydroxyapholide; 8-Methoxyl-14-deoxy-17-β-hydroxyandrographolide; Skullcapflavone I 2′-glucoside; 19-[(β-D-Glucopyranosyl)oxy-19-oxo-ent-labda-8 (17),13-dien-16,15-olide; 14-Deoxy-11,12-didehydroandrographolide; 3,4-Dicaffeoylquininc acid; 5,4′-Dihyfroxy-7-methoxy-8β-D-glucopyranosyloxyflavone; Andrographolide; 19-Hydroxy-3-oxo-ent-labda-8 (17),11,13-trien-16,15-olide; Andrographidine A; Andrographidine E; Androgaraphin; 5-Hydroxy-7,2′,3′-trimethoxyflavone; Dehydrandrographolide l; 14-Deoxy-12-methoxy-andrographolide; Sltosterol; Dihydroskullcapflavone I; Andrographatoside				
Aqueous extract					
Aqueous extract	• Alkaloids	Powdered dried leaves	Evaluation of anti-inflammatory activity of the aqueous extract of *A. paniculata*	N/A (*in vitro*)	Sundar et al. (2015)
	• Flavonoids				
	• Terpenoids				
	• Steroids				
	• Phenols				
Aqueous extract	• andrographolide (AP)	Powdered dried leaves	Suppression of inflammatory cytokines and oxidative stress mediators	5,000 mg/kg in rats (*in vivo*)	Sani et al. (2019)
	• neoandrographolide • 14-deoxy-11,12-didehydroandrographolide				
Other type of extracts					
(1)	*A. paniculata* tablet contains 110 mg lactones which comprise	N/A	Pharmacokinetic study of andrographolide (AP), dehydroandrographolide, neoandrographolide	1 tablet, p.o. in beagle dogs (*in vivo*)	Xu et al. (2015)
	• andrographolide (AP) = 45.0%				
	• dehydroandrographolide = 6.36%				
	• neoandrographolide = 20.0%				
(2)	andrographolide (AP)	Powdered dried leaves	Tissue distribution in rats	133.33 mg/kg/day, p.o. in rats	Bera et al. (2014)
(3)	• andrographolide (AP) = 5%	N/A	Determination of six components of *Andrographis paniculata* extract and one major metabolite of andrographolide in rats	750 mg/kg, p.o. in rats (*in vivo*)	Wang et al. (2014)
	• neoandrographolide = 3.1%				
	• 14-deoxy-11,12-didehydroandrographolide = 3.4%				
	• 14-deoxyandrographolide = 0.4%				
	• chlorogenic acid = 1.9%				
	• apigenin-7-O-β-d-glucuronopyranoside = 1.0%				
Crude powder					
	350 mg/capsule containing	Powdered dried leaves	Pharmacokinetic study of four major diterpenoids	4 capsules, 3 times a day, p.o. (Clinical study)	Pholphana et al. (2016)
	• andrographolide (AP) = 8.16 mg				
	• 14-deoxy-11,12-didehydroandrographolide = 1.35 mg				
	• neoandrographolide = 0.90 mg				
	• 14-deoxyandrographolide = 0.96 mg				

As it is the most bioactive component of *A. paniculata*, andrographolide was identified as a marker, which is of interest for its proven pharmacological activities (Biró-Sándor, 2014), and is mostly analyzed as a major constituent in crude extracts or extract fractions (Naik and Hule, 2009). Therefore, the amount of andrographolide has been used in determining the appropriate dosage of orally administered *A. paniculata* preparations. This review, therefore, compares the pharmacokinetics data in preclinical and clinical studies based on the dosage of andrographolide (Tables 2, 3).

**TABLE 2:** Preclinical and clinical pharmacokinetic studies of *A. paniculata* extract and its bioactive component, andrographolide (AP).

Study type	Type of extract	Formulation	Dose of administration	Dose of AP (as calculated)	Single/multiple dose	Sample size	Model of PK determination	Findings in PK parameters	References
Pharmacokinetics in rats	60% ethanolic water-soluble extract	0.2 g/100 ml	1 mg/kg, i.v.	1 mg/kg, i.v.	single dose	n = 139	One-compartment model	AP is rapidly absorbed in the gastrointestinal tract. This compound is eliminated partially through urine; however, renal excretion is not the main route of AP elimination.	Panossian et al. (2000)
		0.2 g/100 ml	20 mg/kg, p.o.	1 mg/kg, p.o.					
		2 g/100 ml aqueous solution	200 mg/kg, p.o.	10 mg/kg, p.o.					
Pharmacokinetics in humans	fixed combination (equal to 20 mg of AP)	1 tablet	4 tablets	20 mg/day	single dose	n = 16	Two-compartment model		
Pharmacokinetics in rats	andrographolide (Purity >98%)	solution in 20% (w/w) β-cyclodextrin aqueous solution	24 mg/kg, i.v.	24 mg/kg, i.v.	single dose	n = 5	Non-compartmental pharmacokinetics	The absolute bioavailability of AP in rats is found to be 2.67%. This compound is transported to the small intestine and biotransformed there. The metabolism process of AP occurs in the liver as well, which is probably a reason for its poor oral bioavailability.	Ye et al. (2011)
			120 mg/kg, p.o.	120 mg/kg, p.o.					
Pharmacokinetics in rats	andrographolide	Solution in 0.5% w/v SCMC solution in water containing 0.025% v/v tween 80	30 mg/kg/day, p.o.	30 mg/kg/day	single dose	n = 4	Non-compartmental pharmacokinetics	The distribution of AP is unrestricted since the compound can reach several organs (including liver, lung, kidney, heart, and spleen) within 1 h, and can remain in these tissues until 8 h after dosing.	Bera et al. (2014)
Tissue distribution in rats	extract		133.33 mg/kg/day, p.o.	N/A	multiple doses (4 weeks)	n = 48 (24/group)			
	andrographolide		100 mg/kg/day, p.o.	100 mg/kg/day					
Bioavailability in rabbits	aqueous extract	aqueous solution	7.04 ml/kg, p.o.	N/A	single dose	n = 14	N/A	AP is absorbed in the stomach (Tmax is observed at 1 h after oral administration), then AP is distributed to blood circulation and finally biotransformed in the liver.	Levita, (2014)
Pharmacokinetics in beagle dogs	*A. Paniculata* tablet	tablet containing 45.0% of AP	1 tablet, p.o.	49.5 mg for AP	single dose	n = 5	Non-compartmental pharmacokinetics	Tmax is observed at 1.30 h, which is close to this parameter in humans.	Xu et al. (2015)
Determination of metabolites in rats	aqueous ethanolic extract	extract containing 6.5% of AP	750 mg/kg, p.o.	48.75 mg/kg	single dose	n = 6	Non-compartmental analysis	Main hydrolysis metabolites of AP can be found in the circulation system in rats	Wang et al. (2014)
Comparative pharmacokinetics in rats	andrographolide (purity >98.5%)	solution	5 mg/kg, i.v.	5 mg/kg	Single dose	n = 6	N/A	14-deoxy-12-hydroxy-andrographolide, a phase one metabolite of AP, was identified in rat serum. Pharmacokinetic characteristics of AP and its metabolite are significantly different.	Yang et al. (2013)
Metabolism in rats	ethanolic extract	aqueous solution	80 g/kg, p.o.	N/A	single dose	n = 6	N/A	14 tentative metabolites are identified in rat urine and feces, with further speculation on their metabolic pathways.	Li et al. (2015)
Identification of metabolites in rats	andrographolide	N/A	120 mg/kg, p.o.	120 mg/kg	single dose	N/A	N/A	The main metabolite of AP is determined as 14-deoxy-12(R)-sulfo-andrographolide	He et al. (2003b)
Identification of metabolites in rats	extract	aqueous solution	120 mg/kg, p.o.	N/A	N/A	N/A	N/A	Six new andrographolide metabolites are detected in rat urine and feces. Three of them are sulfate esters identified as new metabolites of AP.	He et al. (2003a)
Pharmacokinetics in healthy volunteers	Crude powder	350 mg/capsule containing 8.16 mg of AP	4 capsules, 3 times a day, p.o.	97.92 mg/day	multiple doses (3 consecutive days)	n = 20	Non-compartmental analysis	The four major bioactive diterpenoids: (1) andrographolide, (2) 14-deoxy-11, 12-didehydroandrographolide, (3) neoandrographolide, and (4) 14-deoxyandrographolide were analyzed in plasma. The pharmacokinetic parameters were indicated for each compound.	Pholphana et al. (2016)
Identification of metabolites in healthy volunteers	andrographolide (purity >98.5%)	tablet containing 50 mg of AP	3 tablets, 3 times a day, p.o.	450 mg/day	multiple doses (2 consecutive days)	n = 8	N/A	The possible metabolic pathway of AP is associated with phase II conjugation, including cysteine S-conjugate, sulfate conjugates, and glucuronide conjugates. These conjugate metabolites can be detected in human urine.	Cui et al. (2004), Cui et al. (2005), Qiu et al. (2012), Cui et al. (2008)

Abbreviation

SCMC, sodium-carboxymethyl cellulose.

AP, andrographolide.

**TABLE 3:** Pharmacokinetic parameters of andrographolide (AP) in preclinical and clinical studies.

PK parameters	Pharmacokinetic studies (PK) [Reference]						
	PK in rats Ref: Panossian et al. (2000)	PK in rats^a^ Ref: Panossian et al. (2000)	PK in rats^a^ Ref: Panossian et al. (2000)	PK in rats^a^ Ref: Panossian et al. (2000)	PK in rats^a^ Ref: Panossian et al. (2000)	PK in rats^b^ Ref: Ye et al. (2011)	PK in rats^b^ Ref: Ye et al. (2011)
Route of administration	i.v.	p.o.	p.o.	p.o.	p.o.	p.o.	i.v.
Dose of AP	1 mg/kg	1 mg/kg	10 mg/kg	1 mg/kg	10 mg/kg	120 mg/kg	24 mg/kg
Model of PK	N/A	Independent PK	Independent PK	One-compartmental analysis	One-compartmental analysis	Non-compartmental analysis	Non-compartmental analysis
C_max_	N/A	1.27 ± 0.20 (µg/L)	3.00 ± 0.60 (µg/L)	1.27 ± 0.20 (µg/L)	3.00 ± 0.60 (µg/L)	0.23 ± 0.05 (µg/ml)	N/A
T_max_	N/A	2.41 ± 0.15 (h)	1.67 ± 0.30 (h)	2.41 ± 0.15 (h)	1.67 ± 0.30 (h)	29.75 ± 0.50 (min)	N/A
AUC_0-∞_	7.92 (µg.h/ml)	7.09 ± 1.55 (µg.h/ml)	15.07 ± 2.00 (µg.h/mL)	7.32 ± 1.01 (µg.h/ml)	14.94 ± 2.10 (µg.h/ml)	29.45 ± 3.73 (µg/min ml)	220.83 ± 18.26 (µg/min ml)
Vd	0.27 (L)	N/A	N/A	0.04 ± 0.01 (L)	0.06 ± 0.10 (L)	N/A	24.13 ± 9.63 (L/kg)
Cl	0.24 (ml/min)	0.29 ± 0.07 (ml/min)	0.35 ± 0.22 (ml/min)	0.26 ± 0.04 (mL/min)	0.36 ± 0.50 (ml/min)	109.24 ± 8.99 (mL/min·Kg)	109.24 ± 8.99 (mL/min·Kg)
t_1/2_	1.31 (h)	2.40 ± 0.40 (h)	2.90 ± 0.80 (h)	3.10 ± 0.20 (h)	2.54 ± 0.10 (h)	t_1/2 α_ = 7.92 ± 1.91 (min)	t_1/2 β_ = 157.60 ± 74.09 (min)
						t_1/2 β_ = 142.30 ± 34.48 (min)	
K_el_	0.53 (h^−1^)	0.34 ± 0.15 (h^−1^)	0.31 ± 0.13 (h^−1^)	0.22 ± 0.02 (h^−1^)	0.27 ± 0.01 (h^−1^)	N/A	N/A
MRT	2.9 (h)	4.71 ± 0.70 (h)	4.78 ± 1.14 (h)	5.54 ± 0.10 (h)	4.32 ± 0.20 (h)	N/A	N/A
F	N/A	0.91	0.21	0.91	0.21	2.67%	N/A

PK parameters	Preclinical pharmacokinetic studies (PK)[reference]				Clinical pharmacokinetic studies (PK) [reference]	
	PK in rats^a^ Ref: Bera et al. (2014)	PK in rabbits Ref: Levita (2014)	PK in dogs^a^ Ref: Xu et al. (2015)	PK in rats^b^ Ref: Yang et al. (2013)	PK in humans^a^ Ref: Panossian et al. (2000)	PK in humans^a^ Ref: Pholphana et al. (2016)
Route of administration	p.o.	p.o.	p.o.	i.v.	p.o.	p.o.
Dose of AP	30 mg/kg/day	N/A	49.5 mg of AP	5 mg/kg	20 mg of AP	97.92 mg/day
Model of PK	Non-compartmental analysis	N/A	Non-compartmental analysis	N/A	Independent PK	Non-compartmental analysis
C_max_	115.81 ± 17.56 (ng/ml)	0.2136 (µg/ml)	209.40 ± 64.45 (µg/ml)	N/A	N/A	32.41 ± 3.38 (ng/ml)
T_max_	0.75 ± 0.29 (h)	1.5 (h)	1.30 ± 0.57 (h)	N/A	1.36 ± 0.08 (h)	0.78 ± 0.08 (h)
AUC_0-∞_	278.44 ± 28.64 (ng.h/ml)	0.434 (µg.h/ml)	525.29 ± 196.39 (µg.h/ml)	56.11 ± 14.16 (µg.min/ml)	1,294.00 ± 121.20 (µg.h/ml)	55.23 ± 6.78 (ng^.^h/ml)
Vd	N/A	N/A	300.06 ± 74.85 (L)	53,649.81 ± 16,340.66 (ml/Kg)	55.16 ± 11.81 (L)	Vd/F = 2,712.94 ± 611.64 (L)
Cl	N/A	N/A	106.42 ± 43.46 (L/h)	94.99 ± 29.02 (ml/min/Kg)	271.30 ± 27.58 (ml/min)	Cl/F = 762.51 ± 91.93 (L/h)
t_1/2_	2.45 ± 0.44 (h)	N/A	2.08 ± 0.99 (h)	t_1/2 α_ = 14.82 ± 9.54 (min)	3.94 ± 1.06 (h)	2.65 ± 0.47 (h)
				t_1/2 β_ = 403.19 ± 108.84 (min)		
K_el_	0.29 ± 0.06 (h^−1^)	N/A	N/A	N/A	N/A	0.40 ± 0.06 (h^−1^)
MRT (h)	N/A	N/A	N/A	440.95 ± 102.73 (min)	5.29 ± 1.31 (h)	1.72 ± 0.10 (h)
F	N/A	N/A	N/A	N/A	N/A	N/A

a **Notes**: Data are expressed as mean ± SE.

b Data are expressed as mean ± SD.

Abbreviations: Cmax, maximum plasma concentration; Tmax, time to reach maximum concentration; AUC_0-∞_, area under the plasma concentration–time curve from time zero to the last quantifiable concentration and extrapolated to time infinity; Vd, volume of distribution; Vd/F, the apparent volume of distribution; Cl, clearance; Cl/F, the apparent clearance; t_1/2_, elimination half-life; t _1/2 α_, distribution half-life in alpha phase; t_1/2 β_, elimination half-life in beta phase; K_el_, elimination rate constant; MRT, mean resident time; F, bioavailability.

## 4 Contribution of *Andrographis paniculata* against SARS-CoV-2

### 4.1 *In silico* studies

A computational study has recently investigated the antiviral activities of AP by using bio- and chemoinformatics. The results indicated that AP can be considered to have anti-SARS-CoV-2 activity because it inhibits proteinase activity in the same manner as lopinavir, a drug of choice for the treatment of COVID-19 (Pitakbut, 2020). Recently, an *in silico* study has predicted the possible effects of natural compounds responsible for the anti-SARS-CoV-2 activity. The study suggested that bioactive compounds of *A. paniculata* have shown good binding affinities with SARS-CoV-2 target proteins, including S protein, 3CLpro, PLpro, and RdRp (Hiremath et al., 2021). The binding affinity of APE was found to be lower than −6 kcal/mol, which is considered a useful cut-off point to indicate a compound’s potential as a therapeutic drug candidate (Hiremath et al., 2021). Taken together, molecular docking analysis indicated that AP could enter the binding pocket of the main protease (Mpro) of SARS-CoV-2, an enzyme required for viral replication (Shi et al., 2020). The Mpro is, therefore, considered a promising target protein for drug therapies against COVID-19 (Ullrich and Nitsche, 2020). In a systems biology study, AP showed high levels of interaction with several target proteins of SARS-CoV-2. The analysis of network pharmacology showed that the compound can regulate chemokine signaling, Rap1 signaling, cytokine–cytokine receptor interaction, MAPK signaling, NF-kappa B signaling, RAS signaling, p53 signaling, HIF-1 signaling, and natural killer cell-mediated cytotoxicity (Khanal et al., 2021). A network analysis integrated with experimental validation has also been carried out on the anti-inflammatory effects of *A. paniculata*. The study revealed that IL-6, VEGFA, PTGST2, TNF-α, and MMP-9 were targets of *A. paniculata* in the treatment of inflammation (Zhu et al., 2021). Angiotensin-converting enzyme 2 (ACE-2) has been identified as one of the main receptors to which the SARS-CoV-2 virus binds (Bourgonje et al., 2020). A previous *in silico* prediction found that a possible mechanism of *A. paniculata*’s beneficial effects could involve the inhibition of this receptor (Lim et al., 2021). In addition, this study has predicted the binding of AP to antiviral targets, including the spike protein-ACE-2 receptor complex, spike protein, ACE-2 receptor, RdRp, 3CLpro, PLpro, and N-protein RNA-binding domain (Lim et al., 2021). The findings in these *in silico* studies imply that AP possesses multi-pathway targets contributing to its SARS-CoV-2 antiviral properties.

### 4.2 *In vitro* studies

*In vitro* post-infection treatment of human lung epithelial cells with either APE or its major component AP, has shown that both possess pharmacological activity against SARS-CoV-2 infection in a dose-dependent manner (Sa-Ngiamsuntorn et al., 2021). The study highlights not only the benefits of this plant against coronavirus, but also its favorable cytotoxicity profiles in human liver (HepG2 and imHC), kidney (HK-2), intestine (Caco-2), lung (Calu-3), and brain (SH-SY5Y) cell lines. Both tested compounds showed strong antiviral activity in Calu-3 cells with IC_50s_ of 0.036 μg/ml and 0.034 µM, for APE and AP, respectively (Sa-Ngiamsuntorn et al., 2021). Compared to standard treatment, those IC_50s_ are nearly equivalent to that of the antiviral drug, remdesivir (IC_50_ = 0.086 µM), as determined by a plaque assay in the post-infectious phase. The study indicated that APE and AP mainly affected the late stages of viral replication (Kanjanasirirat et al., 2020; Sa-Ngiamsuntorn et al., 2021). Recently, the SARS-CoV-2 main protease (Mpro), a key enzyme in the viral replication cycle, has been proposed as a potential drug target for COVID-19 therapeutic intervention (Ziebuhr, 2004; Pillaiyar et al., 2016; Chen et al., 2020). This *in vitro* study indicates that AP inhibits the Mpro activity of SARS-CoV-2, and thus, is likely to interfere with the replication of the virus (Shi et al., 2020). This finding supports the selection of *A. paniculata* and AP as candidates for the treatment of SARS-CoV-2 infection.

### 4.3 Clinical studies

Clinical studies have continued to assess the effectiveness of *A. paniculata*. A study was conducted to focus on the use of the extracts of this plant as part of combination therapy, alongside standard supportive care for COVID-19 (Zhang X.-Y. et al., 2021). The result showed that patients with mild to moderate symptoms, who received standard supportive care and a water-soluble AP sulfonate, were less likely to have their infection progress to the severe stage when compared to patients who received only standard treatment. In this study, AP sulfonate was injected as an intravenous (i.v.) fluid drip at a dosage of 10 mg/kg/day (maximum dose 500 mg/day) for 7–14 days and the patients were followed up until 28 days. There were no serious adverse events reported during the study. The safety and efficacy data offer further support for using AP in the treatment of patients with mild to moderate COVID-19 (Zhang X.-Y. et al., 2021).

In addition, a pilot study on six Thai patients who had a positive COVID-19 test with mild symptoms was conducted during the pandemic in Thailand (Benjaponpitak et al., 2021). This study found that APE treatment (equivalent to 180 mg/day), along with standard supportive therapy for 5 consecutive days, was associated with a significant reduction in the severity of COVID-19-related cough and headache (*p* < 0.05) during days 3 and 5 of the study. On day 5, three patients had negative COVID-19 RT-PCR test results (Benjaponpitak et al., 2021). Regarding COVID-19-associated inflammation mediated by cytokines, a further study reported that the levels of adaptive and pro-inflammatory cytokines, growth factors, and chemokines were reduced. This finding supported further investigation of this co-treatment approach in a larger group of patients (n = 309) with mild COVID-19. The study found that combination therapy with APE was effective in relieving symptoms in 306 of the volunteers by day 5. Only three patients (0.97%) in this group developed pneumonia (Benjaponpitak et al., 2021). Therefore, co-treatment with standard supportive care and *A. paniculata* extract may reduce the severity of symptoms in patients with mild COVID-19 and shorten their treatment duration.

## 5 Pharmacokinetics/disposition kinetics reviews

Pharmacokinetics is defined as the study of a drug’s or xenobiotic’s movements through the body’s processes over time. These movements are considered in terms of absorption, distribution, metabolism, and elimination (ADME) (Saghir and Ansari, 2018). The reviews of preclinical and clinical pharmacokinetics of *A. paniculata* are summarized in Table 2 and the pharmacokinetic parameters are shown in Table 3.

### 5.1 Preclinical pharmacokinetics after oral dosing in animals

#### 5.1.1 Absorption

Absorption is a process by which the administered substances enter the blood circulation from the site of administration. Studies on the absorption phase of orally administered APE in rats demonstrated that AP was mainly absorbed in the gastrointestinal tract (Panossian et al., 2000). However, the maximum plasma concentration (Cmax) and the area under the curve (AUC_0-∞_) were observed to be very low (Panossian et al., 2000). This is possibly a result of its poor oral bioavailability, which can be explained by its chemical properties. AP possesses moderately specific lipophilicity (log *p* = 2.632) (Pandey and Rao, 2018), probably leading to a restricted ability in passing this compound through the circulation system. The absolute bioavailability in rats was reported at 2.67% when orally administered at 120 mg/kg of pure AP (Ye et al., 2011). In addition, another animal pharmacokinetic study indicated that orally administered APE (60% ethanolic extract) at doses of 20 mg/kg and 200 mg/kg showed different bioavailability at F = 0.91 and 0.214, respectively (Panossian et al., 2000). The different bioavailability between the two studies is likely due to their use of different types of AP (single compound vs*.* extract). Other constituents in the extract can have a synergistic effect on the pharmacokinetics of AP, which then enhances its bioavailability.

Regarding the pharmacokinetic parameters of AP (Table 3), a previous study on rats reported that plasma levels of this compound reached their maximum concentrations within 0.75 h (Tmax) after receiving 30 mg/kg, p. o. of AP (Bera et al., 2014). This value is close to the Tmax reported in another study, which was reached within 0.5 h when 120 mg/kg of pure AP was orally administered (Ye et al., 2011). Administration of a higher dose of ethanolic APE contributed to faster absorption, since the Tmax observed in a group taking 200 mg/kg was less than the Tmax observed in a group taking 20 mg/kg of APE (1.67 h vs*.* 2.41 h) (Panossian et al., 2000). The influence of interspecies differences on pharmacokinetics, in particular the absorption process, was further investigated in rabbits (Levita, 2014; Mutakin et al., 2020) and revealed that the Tmax occurred at 1 h after oral administration of the extract (Levita, 2014). Moreover, the study was also carried out in beagle dogs, a non-rodent animal that is commonly used in pharmacological studies because of having a gastrointestinal tract more similar to humans (Kararli, 1995). The Tmax was observed at 1.30 h following oral administration of *A. paniculata* tablets containing 49.5 mg of AP (Xu et al., 2015). These results point to the species-specific absorption rates, with the Tmax ranging from 0.5 to 2.41 h in different animal species.

In comparing the Cmax parameter in rats receiving different doses (20 mg/kg and 200 mg/kg) of APE, the Cmax of the group that received the higher dose was approximately 2-fold greater than the Cmax of the group receiving the lower dose (Table 3). It should be noted that increasing the orally administered dose of APE by 10-fold did not cause a 10-fold increase in the Cmax and AUC_(0-∞)_ (Panossian et al., 2000). This observation implies either that AP does not have a dose-dependent impact or that the systemic exposure to this substance may be limited.

#### 5.1.2 Distribution

Following the oral administration of APE, the pharmacokinetic profiles indicated that AP partially enters the blood circulation system. Then, the compound readily penetrates across the cell membrane and is, therefore, widely distributed throughout the body (Panossian et al., 2000). A study on distribution was undertaken in which rats received APE calculated to deliver 100 mg/kg/day of AP (Bera et al., 2014). The study found that the distribution of AP is unrestricted as AP reached multiple organs including the liver, lungs, kidneys, heart, and spleen within 1 h and it remained in these organs until 8 h after dosing (Bera et al., 2014). The existence of AP in lung tissue may indicate an advantage in the treatment of COVID-19 since the lung is one of the target organs most affected by SARS-CoV2 infection (Gavriatopoulou et al., 2020). Moreover, a small amount of AP was detected in the rat brain 1–3 h post-dose (Bera et al., 2014), indicating that some of the compounds could possibly be crossing the blood–brain barrier, a highly selective semipermeable membrane that strictly limits the substances passing into the brain. As mentioned in a previous study, the evidence indicates that AP exhibits neuroprotective effects in the central nervous system (CNS) (Lu et al., 2019). A previous *in silico* study predicted the ability of AP to inhibit ACE-2. AP’s distribution and localization in the brain might indicate that this compound binds to and inhibits ACE-2 in the brain (Bourgonje et al., 2020). To better understand the mechanisms of AP, further investigation into this process is warranted.

The distribution parameter was measured in terms of the volume of distribution (Vd), as shown in Table 3. The Vd of AP was reported to be between 0.04–0.06 L in a one-compartment model (Panossian et al., 2000). A large Vd, calculated by non-compartmental analysis, was found in beagle dogs after receiving *A. paniculata* tablets (Xu et al., 2015). The distribution kinetics of each compound can be significantly influenced by the degree of protein binding in the circulatory system given that only the free form can pass through biological membranes and reach the target organs. As mentioned in the *in vitro* evaluation of the protein binding section, the evidence indicates that AP binds with human plasma proteins with approximately 60% of circulating AP bound to proteins. This means that 40% of the total dose is available to be transported into the specific target locations and produce the desired pharmacological effects (Panossian et al., 2000).

#### 5.1.3 Metabolism

Metabolic pathways are mainly categorized as either phase I or phase II pathways. The most common drug modification in phase I is hydroxylation, a pathway that converts the parent compound to a more polar metabolite. The study by Wang et al. (2014) identified a possible hydrolysis metabolite of AP that could be found in the systemic circulation in rats (Wang et al., 2014). Yang et al. (2013) characterized a possible metabolite, 14-deoxy-12-hydroxy-andrographolide, in phase I metabolism (Yang et al., 2013). In addition, this reaction is also facilitated by enzymatic conversions, particularly in the oxidation and reduction processes, which are catalyzed by cytochrome (CYP) P450 enzymes. Members of this superfamily of enzymes can be induced or inhibited by other substances, leading to an unanticipated adverse event or inappropriate therapeutic response. The study on rats by Zhang et al. (2018) indicated the potential for adverse herb–drug interactions due to substances possessing CYP-modulating properties, especially with warfarin (an anticoagulant medication). The results showed a significant effect due to increases in the plasma levels of warfarin when orally co-administered with AP, thus changing the pharmacokinetic profile of warfarin (Zhang et al., 2018). This finding indicates that AP likely inhibited CYP450 type 3A4 and 2C9 activities, thereby decreasing the rate of warfarin metabolism (Zhang et al., 2018). This interaction is supported by an *in vitro* study that investigated the modulatory influence of APE on CYP450 enzymes (Pan et al., 2011). The study reported a significant weak-to-moderate inhibitory effect on CYP3A4 isoform activity. An aqueous extract weakly inhibited CYP2C9, CYP2D6, and CYP3A4, while ethanolic extracts exhibited higher degrees of inhibition (Pan et al., 2011). A further investigation of the interactive effects of combination therapy on cytochrome P450s found that APE and fluorouracil (5-FU) could reduce the levels of CYP2D6 in human hepatocellular carcinoma (HepG2) cells (Suriyo et al., 2021). Therefore, drug–herb interactions should be taken into consideration when co-administering APE with chemotherapy.

In phase II conjugation, a reaction often occurs to increase the molecular weight by adding a polar moiety such as glucuronate, acetate, and sulfate. This polar moiety increases the water solubility of the substance. A comprehensive report on the metabolism of orally administered *A. paniculata* suggested that AP is extensively metabolized, resulting in detectable metabolites in rats. A total of 14 metabolites were identified in rat urine and feces using an LC/QTOF-MS technique, and the possible metabolic pathways giving rise to them were proposed (Li et al., 2015). A study by He et al. (2003b) also identified the main metabolite of AP, which was determined to be 14-deoxy-12(R)-sulfo andrographolide (He et al., 2003b). Six other metabolites were identified, including three that were categorized as sulfate ester compounds: **(1)** 14-deoxy-12(R)-sulfo andrographolide 3-sulfate, **(2)** 14-deoxy-12(S)-sulfo andrographolide 3-sulfate, and **(3)** 14-sulfo isoandrographolide 3-sulfate. The remaining three identified metabolites were **(4)** 14-deoxy-11,12-didehydroandrographolide, **(5)** isoandrographolide, and **(6)** 14-deoxy andrographolide (He et al., 2003a).

Additional data on the metabolism of AP in rats illustrate that sulfate metabolites are found in the liver within 2 h after oral administration of 120 mg/kg of APE (Ye et al., 2011). The concentration of this metabolite in the liver was 20-fold higher than its concentration in plasma (Ye et al., 2011). This finding indicates an accumulation of the compound in the rats’ livers (Ye et al., 2011; Bera et al., 2014), implying that the compound undergoes biotransformation there. These data strengthen the previous hypothesis that sulfation is likely one of the major metabolic pathways for AP biotransformation in rats (Ye et al., 2011). In addition, other research studies indicate that the compound is metabolized not only in the liver but also in the intestine, including the duodenum, jejunum, and probably the terminal ileum or colon as well (Mehta et al., 2021). This finding involves the effect of first-pass metabolism, which reflects its low oral bioavailability, decreasing the concentration of AP before it enters the blood circulation (Ye et al., 2011). The process of metabolism is, therefore, considered to be one reason for its poor bioavailability. Another minor contributor to the low bioavailability could be the expression of P-glycoprotein (P-gp), an efflux transporter, which limits the cellular uptake of xenobiotics. The effect of the competition between AP and other drugs on P-gp-mediated efflux needs to be considered, as co-administration with other medicines could possibly result in a drug interaction that could change the pharmacokinetics and pharmacological therapeutic effects (Mehta et al., 2021).

#### 5.1.4 Elimination

In the termination phase, AP is eliminated in both its unchanged and conjugated forms. Findings in preclinical pharmacokinetic studies suggest that AP undergoes a process of metabolism, resulting in the generation of metabolites that are eliminated, in part, through urinary excretion (Ye et al., 2011; Bera et al., 2014). The report on rat metabolism of isolated AP demonstrated that sulfate-conjugated metabolites are finally excreted by renal elimination (Ye et al., 2011). This finding is supported by other evidence pointing to the accumulation of AP in the kidneys from 1–8 h after oral administration (Bera et al., 2014), which is highly suggestive of renal excretion.

Focusing on the pharmacokinetic parameters, when comparing the lower dose (20 mg/kg, p. o.) and a 10-fold higher dose (200 mg/kg, p. o.) of APE, the elimination half-life (t_1/2_) was consistent at approximately 2–3 h in both groups. Renal clearance parameters (Cl_r_), however, were observed to be different between those two dosages, with an increased Cl_r_ value in the higher dose group (Panossian et al., 2000). In addition, the study on rats showed that AP was extensively eliminated within 6 h after receiving 20 mg/kg of APE. The highest amount of AP was eliminated through urine, which accounted for only 7–9% of the administered dose. Therefore, more than 90% of this compound was presumably excreted via other pathways. For the elimination of higher doses, the group that received 200 mg/kg showed a longer elimination interval of 6–24 h, with renal excretion, since 0.5% of AP could be eliminated via urine. Interestingly, the elimination rate constant (k_el_) also demonstrated a higher value in the low-dose group (Panossian et al., 2000). This finding in the termination phase may indicate that AP is probably eliminated partly through renal excretion.

### 5.2 Clinical pharmacokinetics after oral administration in healthy volunteers

#### 5.2.1 Absorption

The findings concerning the absorption phase of AP in human pharmacokinetics are consistent with the data in animal studies. As indicated by the clinical pharmacokinetic parameters, the compound was rapidly absorbed and reached a maximum plasma concentration at 1.6 h after tablets containing 200 mg of APE were administered to healthy Chinese volunteers (n = 20) (Xu et al., 2009). A study applying the two-compartment model found that the Tmax occurred within 1.36 h post-dose when participants had received 20 mg of orally administered APE (Panossian et al., 2000). Furthermore, a study on multiple oral dosing, which involved administering an equivalent dose of 97.92 mg/day of AP in healthy Thai volunteers, reported that the Tmax occurred at 0.78 h during a steady state of 3 consecutive days (Pholphana et al., 2016). This study also investigated the pharmacokinetic parameters of other diterpenoids, and the results suggested that the AUC and C_max_ of 14-deoxy-11, 12-didehydroandrographolide were higher than that of AP (Pholphana et al., 2016). The result may suggest the possible effects of andrographolide derivatives responsible for the biological effects of this plant. Further pharmacokinetic studies are recommended to support this finding.

The C_max_ and AUC corresponding to different dosages and different formulations of AP are compared in Table 3. The extent of AP in the blood circulation was observed to be very low, as indicated by the manner in which Cmax and AUC parameters showed minimal values even after high dosages were administered. These findings imply that the compound possesses poor oral absorption, possibly contributing to its low bioavailability, which corresponds to the results from preclinical studies.

#### 5.2.2 Distribution

In human pharmacokinetics studies, the distribution of AP was determined in four subjects using a one-compartment model by the TOPFIT program. The study showed that AP was rapidly distributed to the tissues. Another part of the study was conducted on 12 volunteers and predicted that the distribution would take 1.5–3 h when calculated using the two-compartment model (Panossian et al., 2000). This study also emphasized AP’s ability to bind extensively with plasma proteins since it illustrated high individual pharmacokinetic variability, probably due to the different levels of protein binding in each participant. This finding is also supported by evidence of the high binding affinity of AP with plasma proteins in the *in vitro* study (Panossian et al., 2000).

To investigate the possibility that AP taken by a pregnant patient could be distributed to the fetus, various modulating factors affecting diffusion across the placenta must be taken into consideration. Non-ionized molecules with molecular weights less than 600 Da that exhibit hydrophilic chemical properties can pass through the placenta by passive diffusion (Rubinchik-Stern and Eyal, 2012). Although AP is an un-ionized lipophilic molecule of 350.4 g/mol molecular weight, it still demonstrates high-affinity protein binding and its bound form is unable to move across the placenta. The proportion of protein binding, however, can be altered by pathological conditions. Therefore, the use of AP in pregnant patients should be evaluated on a case-by-case basis, taking into account the individual’s relevant health factors.

#### 5.2.3 Metabolism

A previous study investigated the possible metabolites of AP in human urine and identified the conjugated metabolites in phase II metabolism. Urine samples were collected from healthy volunteers from 0 to 72 h after the volunteers received repeated doses calculated as 450 mg of AP per day for 2 consecutive days (Cui et al., 2004). The study identified four possible metabolites in urine by infrared spectroscopy and further characterized their chemical structures by NMR spectroscopy. One of these metabolites was a cysteine S-conjugate, 14-deoxy-12-(cysteine-S-yl)-andrographolide-3-O-sulfate. Three metabolites were sulfate conjugates, namely, **(1)** andrographolide-3-O-sulfate, **(2)** isoandrographolide-3-O-sulfate, and **(3)** 14-deoxyandrographolide-3-O-sulfate (Cui et al., 2004). Subsequently, this research group continued their work on metabolite identification related to phase II metabolism and compiled additional data on glucuronide conjugate forms. These metabolites, which were characterized by LC/MS and NMR spectroscopy, were as follows: **(1)** andrographolide-19-O-beta-D-glucuronide; **(2)** isoandrographolide-19-O-beta-D-glucuronide; **(3)** 14-deoxy-12-hydroxy-andrographolide-19-O-beta-D-glucuronide; **(4)** andrographolide-19-O-[6′-methyl-beta-D-glucuronide]; **(5)** 14-deoxy-12 (13)-en-andrographolide-19-O-beta-D-glucuronide; **(6)** 14-deoxyandrographolide-19-O-beta-D-glucuronide; and **(7)** 3-oxoandrographolide-19-O-beta-D-glucuronide (Cui et al., 2005). The conjugate forms of 19-*O*-glucuronide, which account for more than 80% of the total metabolites in human urine, were considered to comprise the major metabolites of AP (Cui et al., 2005). These findings are consistent with a previous report suggesting that the 19-*O*-glucuronide metabolite exhibits potent anti-inflammatory activities (Tian et al., 2015). Recently, a study also detected creatinine adducts in human urine which are as follows: **(1)** 14-deoxy-12-(creatinine-5-yl)-andrographolide-19-O-βd-glucuronide A and **(2)** 14-deoxy-12-(creatinine-5-yl)-andrographolide-19-O-β-d-glucuronide B, as indicated by MS/MS fragmentation and ^1^H-NMR spectroscopy (Qiu et al., 2012). Later on, another four urea adducts were sequentially reported as possible minor metabolites of AP and these minor metabolites accounted for less than 0.1% of the orally administered dose. However, there is limited information available in the literature about the metabolic pathway(s) responsible for urea adducts (Matsuda et al., 1994; Cui et al., 2008). Therefore, further investigation of the following metabolites is recommended: **(1)** 14-deoxy-12-carbamidoandrographolide; **(2)** 14-deoxy-12-carbamidoandrographolide- 19-O-sulfate; **(3)** 14-deoxy-12 (R/S)-carbamido- andrographolide-19-O-β-D-glucuronide; and **(4)** 14-deoxy-12(S/R)- carbamidoandrographolide-19-O-β-D-glucuronide.

#### 5.2.4 Elimination

Data related to the elimination kinetics of AP in humans are summarized in Table 3. The elimination phase for AP begins within 3–4 h after dosing, and the compound is excreted until 8 h post-dose. The individual elimination half-life (t_1/2_), as indicated by the two-compartment model, was estimated at 2 h after a single oral administration of 20 mg of AP. This t_1/2_ parameter, however, showed a wide range of values across the study participants which was between 2 and 7 h because of the subjects’ variability (Panossian et al., 2000). The study on multiple oral dosing of *A. paniculata* capsules (equivalent to 97.92 mg/day of AP) reported an average t_1/2_ of 2.65 h, when determined via non-compartmental analysis (Pholphana et al., 2016). A multiple-dose study that considered apparent clearance (Cl/F) and included the subgroup analysis by gender found no significant differences between males and females (11.89 vs*.* 13.54 L/h/Kg) with regard to elimination parameters (Pholphana et al., 2016).

When considering the possibility of excretion into breast milk, it is important to keep in mind that the transport of xenobiotics from the mother’s circulation into mammary blood capillaries mostly depends on passive diffusion, lipid solubility, pKa of the compound, and the protein binding of the substance. The pH value of human milk (pH = 7) is slightly lower than that of plasma (pH = 7.4), so weak base compounds tend to be present in breast milk at higher concentrations than in plasma. AP exhibits a pKa of 12.36 and has the physicochemical properties of an un-ionized lipophilic molecule. It would be expected to concentrate in the mother’s plasma and should not be excreted into breast milk. Considering that AP has a high binding affinity to plasma proteins (Panossian et al., 2000), there would also be less of the free form of AP available for transport into breast milk. Although it is unlikely that AP passes into breast milk, the use of this compound in nursing mothers should be discouraged until more is known.

## 6 Present dosage for oral administration of *A. paniculata* in patients with uncomplicated upper respiratory tract infections (URTIs) and other viral infectious diseases

The efficacy of APE and AP was evaluated in clinical studies for expanded usage in the treatment of viral infectious diseases including influenza, herpes simplex virus (HSV), human immunodeficiency virus (HIV), coronavirus (SARS-CoV2), and other URTIs (Jayakumar et al., 2013). This review focuses on the clinical evidence for its effectiveness in the relief of symptoms in uncomplicated URTIs, especially in pharyngotonsillitis, and influenza. The clinical studies on different dosage regimens for the oral administration of APE and its bioactive component, AP, in uncomplicated URTIs and other viral infectious diseases are summarized in Table 4.

**TABLE 4:** Clinical studies of *A. paniculata* extract in uncomplicated upper respiratory tract infections (URTIs) and other viral infectious diseases.

Indication used in clinical trial	Study type	Type of extract/compound	Outcome measurements	Sample size (n)	Administered dose	Duration	Dose of AP (as calculated)	Findings	References
Influenza	An open-label, multicentre, randomized control trial	crude powder 400 mg/capsule (content of total lactones calculated as AP = 9%/capsule)	Body temperature and symptom scores: nasal congestion, sore throat, headache, malaise, myalgia, fatigue, and chill	n = 25	4 capsules, 4 times a day	7 days	576 mg/day	Co-treatment of patients with acetaminophen and A. *paniculata* as early as possible, or within 36 h of the onset of influenza, could reduce the severity of symptoms and shorten their treatment duration.	Chuthaputti et al. (2007)
Acute URTIs	A double-blind, placebo-controlled, parallel-group clinical study	fixed combination (containing 5 mg of AP/tablets)<	Temperature, headache, muscle aches, throat symptoms, cough, nasal symptoms, general malaise, and eye symptoms	n = 200	4 tablets, 3 times a day	5 days	60 mg/day	The total score illustrated a highly significant improvement in the extract group.	Gabrielian et al. (2002)
Uncomplicated acute URTI	A randomized double-blind, placebo-controlled parallel group study	fixed combination (containing 5.25 mg of AP/tablet)	*Patients self-evaluation*: muscle soreness, cough, throat, headache, nasal symptoms, eye signs, and temperature	pilot study: n = 47	4 tablets, 3 times a day	3–8 days	Approximately ≥60 mg/day	*Pilot study*: the total symptom score showed an improvement in the treatment group relative to the placebo group.	Melchior et al. (2000)
			*Physician’s fixed score diagnosis*: symptoms/signs of ears, nose, eyes, oral cavity, and tonsils	phase III: n = 180				*Phase III study*: significant improvement in total symptom score (*p =* 0.0006) and total diagnosis score in the treatment group (*p =* 0.003) as compared with the placebo group.	
Uncomplicated URTI	randomized double-blind, placebo-controlled clinical study	extract containing 30% W/W of AP (1 capsule = 100 mg of extract)	Symptom score evaluated by determining Visual Analog Scale (VAS): cough, expectoration, running nose, headache, fever, sore throat, earache, malaise/fatigue and sleep disturbance	n = 223	1 capsule, twice a day (200 mg/day)	5 days	60 mg/day	Patients receiving the extract showed significant decreases in scores for all symptoms except for earache on day 5.	Saxena et al. (2010)
								This finding indicates that extract of *A. paniculata* is effective in reducing symptoms of URTI.	
Influenza	randomized controlled two parallel group study	fixed combination (containing 5 mg of AP/tablets)	Efficacy parameters: temperature, malaise, headache, rhinitis, pain in throat, cough, conjunctivitis	*Pilot study*: n = 540	*Pilot study*: 2 tablets, 3 times a day	*Pilot study*: 3–5 days	*Pilot study* = 30 mg/day	*A. paniculata* extract was associated with reductions in the duration of disease and decreased risk of post-influenza complications.	Kulichenko et al. (2003)
				*Main study*: n = 66	*Main study*: 3 tablets, 3 times a day	*Main study*: 5 days	*Main study* =45 mg/day		
Pharyngo-tonsillitis	multicentre study	ethanoic extract	*Signs*: temperature, pharynx, tonsillar exudate, pharyngeal petechiae, cervical lymphadenopathy	n = 152	3 capsules, 4 times a day group 1:3 g/day or group 2: 6 g/day	7 days	180 mg/day, 360 mg/day	There was a statistically significant difference in fever and sore throat relief on day 3 relative to day 0 after oral administration of a high dose of *A. paniculata* or acetaminophen.	Thamlikitkul et al. (1991)
		250 mg/capsule	*Symptoms*: sore throat, fever, rhinorrhea, cough, duration of symptoms					Patients were more satisfied with receiving either the higher dose of *A. paniculata* or acetaminophen than receiving a low dose of extract	
		500 mg/capsule (at least 6% of total lactones calculated as AP)	*Concomitant treatment:* antibiotics, antihistamine and/or decongestant, antitussive						
uncomplicated respiratory disease in children (aged 4–11 years)	randomized controlled three parallel group study	fixed combination (containing 5.25 mg of AP/tablet)	*Patient self-evaluation*: pain in muscles, cough frequency, dry cough, productive cough, difficulties in breathing, sore throat, dryness and feeling of roughness in the throat, head-ache, fever, and rhinitis.	n = 133	2 tablets, 3 times a day	10 days	30 mg/day	Receiving *A. paniculata* extract was associated with a significantly decreased level of nasal secretion and nasal congestion as compared to that of other groups in children with common cold.	Spasov et al. (2004)
			*Physician’s fixed score diagnosis*: symptoms/signs of ears, nose, eyes, oral cavity, and tonsils						

In a double-blind placebo-controlled trial, a fixed combination containing standardized extracts from *A. paniculata* and *Eleutherococcus senticosus* was studied in the treatment of acute URTIs (Gabrielian et al., 2002). A total of 200 patients with a diagnosis of URTI were enrolled in this study and were randomly assigned to receive either the fixed combination of APE (calculated as 60 mg/day of AP) or a placebo, for 5 consecutive days. The total score analysis illustrated a significant improvement in the clinical symptoms of URTIs in the treatment group that received APE (Gabrielian et al., 2002).

To corroborate the findings on the efficacy of this fixed combination of the standardized APE 85 mg/tablet (containing 5.25 mg of AP and deoxyandrographolide), a double-blind placebo-controlled (phase III) clinical study was carried out on 180 patients diagnosed with uncomplicated acute URTI (Melchior et al., 2000). The extract (equivalent to 60 mg/day of AP) was given to patients in the treatment group, while participants in the control arm received a placebo for 3 days. The results showed significant improvement in the treatment group not only in terms of the total symptom score (*p* = 0.0006), rating muscle soreness, cough, throat, headache, nasal symptoms, eye signs, and temperature, by the patients, but also in a diagnosis score (*p* = 0.003), evaluating the ears, nose, eyes, oral cavity, and tonsils, by a physician. Furthermore, the number of patients who needed additional medical treatment after day 3 was significantly lower in the treatment group than in the control group. Those patients who received the extract completely recovered from their URTI after days 8–9 (Melchior et al., 2000). These findings demonstrate that this fixed combination extract of *A. paniculata* reduces the common symptoms of uncomplicated URTIs.

To assess the effectiveness of APE in reducing the symptoms of URTIs, a randomized double-blind, placebo-controlled study evaluated a symptom score determined by using a visual analogue scale (VAS) (Saxena et al., 2010). A total of 223 patients with uncomplicated URTIs were randomly assigned to receive either 60 mg/day of AP or a placebo. Comparing the overall symptom scores between day 1 and day 3, the scores in the treatment group were not significantly different from the scores in the placebo group. However, a significant difference was found between the symptom scores of the two groups on day 5. Receiving APE in an amount equivalent to 60 mg/day of AP was associated with a significant decrease in URTI-related symptoms including cough, expectoration, nasal secretion, headache, fever, sore throat, fatigue, and sleep disturbance during days 3–5 (*p* < 0.05) (Saxena et al., 2010).

Clinical research has also been carried out on the use of *A. paniculata* in patients with influenza. An open-label, multicenter, randomized control trial evaluated the effectiveness of *A. paniculata* (crude powder) in alleviating the symptoms of influenza for 7 consecutive days (Chuthaputti et al., 2007). Patients who had a fever ≥38°C, had experienced constitutional symptoms for 36 h or less and had laboratory-confirmed influenza, were recruited and randomized into two groups. One group (n = 10) received 1,000 mg of acetaminophen every 6 hours; the other group (n = 15) received the same acetaminophen treatment and *A. paniculata* capsules (equivalent to a total lactone of 576 mg/day). The study found that body temperature and the severity of symptoms, which included nasal congestion, sore throat, headache, malaise, myalgia, fatigue, and chill, improved significantly by day 2 in the group treated with both acetaminophen and *A. paniculata.* Consistent with the results of the previous study, overall symptoms and the severity of cough and fatigue were decreased by day 4 in the group receiving this co-treatment. There were no significant differences identified between the two groups on days 2, 4, or 6, neither in body temperature nor in other influenza symptoms. This study suggests that combination therapy of acetaminophen and *A. paniculata* may be effective in relieving influenza symptoms within 2 days of the start of treatment. The severity of symptoms was found to lessen by day 2 as compared to day 0. Hence, co-treatment of patients with acetaminophen and *A. paniculata* as early as possible, or within 36 h of the onset of influenza, could reduce the severity of symptoms and shorten their treatment duration (Chuthaputti et al., 2007).

The benefits of using *A. paniculata* during influenza outbreaks were also illustrated by comparing conventional therapy (antiviral agent, acetaminophen, and ascorbic acid) with co-intervention (*A. paniculata* with conventional therapy) (Kulichenko et al., 2003). In the pilot study (first study), patients received a fixed combination of an herbal preparation containing a standardized extract of APE (calculated as 30 mg/day of AP) for 3–5 days. In the main study, they received 45 mg/day of AP for 5 days (second study). Considering the body temperature and clinical symptoms (headache, myalgia, and eye problems), patients receiving *A. paniculata* recovered from these symptoms within 3 days (Kulichenko et al., 2003). Moreover, the group that received co-intervention with *A. paniculata* along with acetaminophen treatment showed a shorter average duration of illness than the group receiving only conventional treatment (7.2 days versus 9.8 days, *p* < 0.001). Both the pilot study and the main study showed that *A. paniculata* was associated with statistically significant decreases in clinical symptoms and reductions in the duration of disease, with decreased risk of post-influenza complications. This finding recommends the fixed combination of standardized APE for use in outpatients during influenza outbreaks (Kulichenko et al., 2003).

In addition, a subgroup analysis was carried out comparing patients with confirmed or unconfirmed cases of the influenza virus (type A). Influenza A virus was identified from the subject’s nasal and throat swabs to classify patients into subgroups for analysis. This study indicated that for confirmed influenza patients that were diagnosed early, conventional treatment with an antiviral agent was more effective compared to the unconfirmed group. Nevertheless, co-treatment with APE was found to shorten the duration of illness in both subgroups, that is, confirmed and unconfirmed patients. This finding suggests that the combination of standard treatment with *A. paniculata* can be applied during influenza outbreaks and used to treat symptoms whether or not influenza is identified in a particular patient (Kulichenko et al., 2003).

To study the use of AP in the treatment of pharyngotonsillitis, a randomized double-blind multicenter study was conducted on 152 Thai patients with pharyngotonsillitis (Thamlikitkul et al., 1991). These volunteers were randomized into three groups to receive either 1) acetaminophen (a potent antipyretic agent), 2) crude powder of *A. paniculata* (calculated as 180 mg/day of AP), or 3) 360 mg/day of calculated AP. The participants were observed for 7 days. This study found that patients who received higher doses of *A. paniculata* or who received acetaminophen could stop their medications on day 3 because they were no longer feverish from day 3 onward (68.1% vs*.* 67.3%) and had completely recovered from their sore throat by that time (29.7% vs*.* 30.6%). There was a statistically significant difference in the relief of fever and sore throat on day 3 for patients who received a high dose of either *A. paniculata* or acetaminophen compared to patients receiving a low dose*.* This significant difference in symptom improvement was not observed on day 7 since all patients in the study had recovered from their illness within a week, even the patients in the low-dose group. The study also evaluated the patients’ satisfaction and the findings suggested that patients were more satisfied with receiving a higher dose of either *A. paniculata* or acetaminophen as than receiving a low dose of the extract (Thamlikitkul et al., 1991). For the safety evaluation, the study showed that nausea, vomiting, abdominal discomfort, dizziness, and malaise were found in 20% of patients in each group; however, these adverse events were considered to be unrelated to the administered drug.

Several clinical studies have also investigated specific populations, particularly children. Spasov et al. (2004) investigated a fixed combination, comprising 85 mg of standardized APE (containing 5.25 mg of AP and deoxyandrographolide) and 9.7 mg of *Acanthopanax senticosus* extract, in children with uncomplicated URTIs (Spasov et al., 2004). A total of 133 subjects, boys and girls aged 4–11 years, were randomized to receive either standard treatment (acetaminophen 500 mg, 3 times a day), co-treatment comprising standard therapy in combination with APE (approximately 30 mg/day of AP), or a combination of standard treatment with Echinacea drops (10 drops, 3 times a day) for 10 days. The results demonstrated that co-treatment with AP (30 mg/day) was safe and well-tolerated in children. Taking APE during the early phase of acute URTIs was associated with a significantly decreased level of nasal secretion and nasal congestion as compared to that of other groups. Children who received APE as adjuvant therapy recovered more quickly from common cold symptoms than children in the other two groups (Spasov et al., 2004).

## 7 Discussion

*A. paniculata* has a long history of use in traditional herbal medicines for URTIs. Its properties have also been investigated through *in silico*, *in vitro*, and *in vivo* studies, and in clinical trials. To determine this plant’s potential for application in the treatment of COVID-19 symptoms, a review of the existing literature is helpful. Therefore, this article compiles the available pharmacokinetics data and proposes recommended dose ranges for the possible use of *A. paniculata* in COVID-19 patients.

According to available evidence on its disposition kinetics, AP is rarely absorbed into the blood circulation with poor oral bioavailability (Ye et al., 2011). Although the absorption of AP seems to be limited after oral administration of APE, studies on its distribution throughout the body showed unrestricted accumulation in organs (Bera et al., 2014). Of particular importance in the context of respiratory ailments, AP can be detected in the lung tissue (Bera et al., 2014). Another relevant aspect of its pharmacokinetics in dose comparisons is that a 10-fold increase in the oral dosage of the extract did not result in 10-fold-greater levels in plasma (Panossian et al., 2000). This indicates that it is dose-independent, possibly due to limitations for its entry into the systemic circulation and intensive metabolism. During phase II metabolism of AP, sulfation (Ye et al., 2011) and glucuronidation (Cui et al., 2005; Cui et al., 2008; Qiu et al., 2012) occur that result in compounds with a greater polarity that are easy to excrete during the termination phase. These changed and unchanged forms of AP are partly eliminated through the hepatobiliary system and also via urinary excretion (Panossian et al., 2000; Cui et al., 2004; Qiu et al., 2012). In considering herb–drug interactions during the metabolism process, concomitant therapy commonly alters pharmacokinetics, in terms of the induction or inhibition of cytochrome P 450 enzymes. This can lead to therapeutic failure and adverse clinical outcomes. Co-administration of APE together with other narrow therapeutic index drugs, particularly warfarin, should be avoided or undertaken with caution. This is because the extract can inhibit CYP2C9, CYP2D6, and CYP3A4, which are the three most important human hepatic enzymes related to herb–drug interactions.

Regarding the appropriate dose range for AP, several studies indicate that orally administered APE, calculated as 60 mg/day of AP, is an effective dose for relieving symptoms in uncomplicated URTIs (Melchior et al., 2000; Gabrielian et al., 2002; Saxena et al., 2010). Therefore, multiple oral administration of AP of at least 60 mg/day is recommended as the starting dose for acute URTI treatment that can be continued for 5–7 consecutive days. Moreover, higher doses of *A. paniculata* (calculated as 180 mg/day or up to 360 mg/day of AP) can reduce symptoms in pharyngotonsillitis patients when it was used for a week (Thamlikitkul et al., 1991).

The evidence gleaned from clinical studies of *A. paniculata* use as a treatment for URTIs can inform its possible application in the treatment of COVID-19 symptoms. The pathophysiology of COVID-19 includes not only URTI-like symptoms but also an inflammatory process after infection with the SARS-CoV-2 virus. Considering the possible anti-inflammatory effects of AP, the reported evidence indicates that AP exhibits anti-inflammatory properties by inhibiting several signaling pathways and interference with different transcription factors (Pandey and Rao, 2018; Burgos et al., 2020). In addition, a recent study also suggested that the metabolic effects of AP are one of the possible pathways associated with its multitarget therapeutic activities (Burgos et al., 2020). Network pharmacology reported that IL-6, VEGFA, PTGST2, TNF-α, and MMP-9 were targets of *A. paniculata* in the treatment of inflammation (Zhu et al., 2021). This consideration supports the use of APE in the treatment of COVID-19 patients (pilot study) with doses equivalent to those used to treat pharyngotonsillitis (180–360 mg/day calculated as AP), an infectious disease that also causes inflammation after viral infection. Therefore, *A. paniculata* calculated to deliver an AP dose of 180 mg/day or up to 360 mg/day, is expected to be effective for use in the co-treatment of the early symptoms of COVID-19. However, a starting dose of 180 mg/day of AP is suggested, according to available safety data. It is recommended to begin the co-treatment within 36 h of symptom onset, to maximize the effect of the drugs’ antiviral mechanism(s) targeting the viral replication stage in the upper respiratory tract and alleviate COVID-19 severity. Evidence shows that receiving APE as early as possible, that is, within 36 h of influenza onset, can reduce the severity of the symptoms within 2 days and shorten the duration of illness (Chuthaputti et al., 2007).

The recommended dosage may vary for different populations, specifically, in children, pregnant women, nursing mothers, and people with certain health conditions. To consider the appropriate dosage for children (Spasov et al., 2004; Hu et al., 2017), a clinical study indicated that a daily dose of APE, calculated to deliver 30 mg of AP, was an effective and well-tolerated dose for children aged 4–11 with uncomplicated URTIs (Spasov et al., 2004). Taken together, the evidence suggests that the AP dose should be adjusted for patients with very low body weight (Pholphana et al., 2016). Therefore, specific dosing in pediatric patients may be calculated according to the individual’s body weight (mg/kg).

When determining the appropriate dose for the use of this medicinal plant in pregnant and lactating women, additional factors should be taken into consideration. The data on the excretion of AP into breast milk is still insufficient; therefore, the recommended dose of this extract for use by nursing mothers is uncertain. Similarly, there is no clear evidence of the potential effects of high doses of *A. paniculata* on liver and kidney functions, and this compound is possibly excreted via both organs as indicated in several reports. Therefore, caution should be taken when this extract is administered to people with hepatic or renal impairment.

## 8 Conclusion

In conclusion, based on the present reviewed pieces of evidence, the administration of a single compound of AP, crude powder, water, or ethanolic extracts, and the standardized extract of *A. paniculata* (calculated as 60 mg/day of AP) is considered to be safe and effective for the treatment of URTIs in adult patients. The recommended dose for patients with mild COVID-19 is determined based on the doses used in patients experiencing a URTI with inflammation. Therefore, a prudent dose for use during COVID-19 outbreaks is recommended at 180 mg/day of AP. The preliminary results in Thailand indicate that this dose of AP can be used alongside standard therapy for COVID-19 treatment. For the most effective results, the optimal time point to start taking *A. paniculata* should be within 36 h of symptom onset or as early as possible. Further research on *A. paniculata* should be carried out to evaluate the appropriate dosages of andrographolide for use during the COVID-19 pandemic.
